# A Review of Current Neuromorphic Approaches for Vision, Auditory, and Olfactory Sensors

**DOI:** 10.3389/fnins.2016.00115

**Published:** 2016-03-29

**Authors:** Anup Vanarse, Adam Osseiran, Alexander Rassau

**Affiliations:** School of Engineering, Edith Cowan UniversityJoondalup, WA, Australia

**Keywords:** neuromorphic sensors, retinomorphic sensors, neuromorphic audition, neuromorphic olfaction, biomimetic sensors

## Abstract

Conventional vision, auditory, and olfactory sensors generate large volumes of redundant data and as a result tend to consume excessive power. To address these shortcomings, neuromorphic sensors have been developed. These sensors mimic the neuro-biological architecture of sensory organs using aVLSI (analog Very Large Scale Integration) and generate asynchronous spiking output that represents sensing information in ways that are similar to neural signals. This allows for much lower power consumption due to an ability to extract useful sensory information from sparse captured data. The foundation for research in neuromorphic sensors was laid more than two decades ago, but recent developments in understanding of biological sensing and advanced electronics, have stimulated research on sophisticated neuromorphic sensors that provide numerous advantages over conventional sensors. In this paper, we review the current state-of-the-art in neuromorphic implementation of vision, auditory, and olfactory sensors and identify key contributions across these fields. Bringing together these key contributions we suggest a future research direction for further development of the neuromorphic sensing field.

## Introduction

The field of neuromorphic engineering has been developing rapidly over the last decade. With the growing trend toward embedding intelligence in day-to-day devices, we are constantly making our surroundings smarter and more adaptive to our behavior. However, this technological progression requires an ever increasing number of sensors and associated data storage (Tenore and Etienne-Cummings, 2011). Along with the data processing challenges, factors such as power consumption and financial viability limit the development of smart devices. Realizing these limitations in the late 1980s, Carver Mead introduced the concept of neuromorphic engineering. This interdisciplinary field addresses the underlying concepts of neurobiological architecture and mimics its implementation using aVLSI. Neurobiological architecture is a low power consuming system which learns through exposure; these attributes, along with sparse output are crucial design criteria for neuromorphic systems (Mead, 1990).

Neuromorphic approaches have been applied in implementing neural processors, developing neural networks, and particularly in electronic sensing where novel methodologies have been developed (Chicca et al., 2014). In the mid 1980s, Max Delbrück, John Hopfield, Carver Mead, and Richard Feynman collaborated to exploit the non-linear current characteristics of transistors (Indiveri and Horiuchi, 2011). Carver Mead, further highlighted the excessive dissipation of energy through conventional computing methods and the limitation of using transistors merely as digital switching components. He proposed that the analog physical properties of transistors could be exploited to design adaptive and low power consuming sensors like silicon retina and cochlea (Mead, 1990). With these models as inspiration, neuromorphic concepts have been applied to vision sensors, auditory sensors, and olfactory sensors. Current sensor advances have been supported by massive parallelism, asynchronous processing and self-organization (Douglas et al., 1995; Liu et al., 2015).

As described in Hasler and Marr (2013), analog implementation of neural-like systems are capable of approaching equivalence to biological systems in terms of power consumption and efficiency. Most of the research in neuromorphic sensors has involved aVLSI; however, with rapidly changing technology, digital electronics has also been applied to implement neuromorphic concepts, particularly as it has proved to be robust to internal and external noise (Sarpeshkar, 1998). Systems implemented using digital electronics are easily programmed and upscaled (Sarpeshkar, 2006). Regardless of whether analog or digital implementations are used, the lack of standards and benchmarks for the output of neuromorphic sensors may limit their development and adoption. In the same way that the interfacing for neuromorphic sensors uses standard Address Event Representation (AER; Boahen, 2000), a standardized method to evaluate sensor outputs could help establish appropriate benchmarks for further improvement. In this paper we review significant recent contributions to neuromorphic vision, auditory, and olfactory sensing and compare them to identify potential benchmarks for neuromorphic sensors.

## Neuromorphic vision sensors

Neuromorphic engineering concepts have been successfully implemented to emulate biological sensory systems with silicon retinas being a prominent example (Tenore and Etienne-Cummings, 2011). Currently, vision sensing depends on the conventional frame-based approach but regardless of whether the scene changes, these frames are captured continuously and this generates significant volumes of redundant data (Lichtsteiner et al., 2008; Brandli et al., 2014). However, reducing the frame capture rate may cause excessive information loss between consecutive frames, particularly for real-time applications such as machine vision and robotics. Such frame-based approaches also consume substantial power and make data management challenging (Posch et al., 2014). Attempts were made to control the data output from these sensors by relaying information only for changed values of pixels. However, off-sensor processing and complex control strategies increased the overall power consumption of the system (Lichtsteiner et al., 2008).

Mahowald and Mead implemented the first silicon retina model in Mahowald and Mead (1991), that was both adaptive and energy efficient, by emulating retina functionalities, especially the cone cells, through analog properties of transistors and introducing adaptive vision sensing., improved this model by adding the functionalities of inner retina and parvo-magno cells (Zaghloul and Boahen, 2004a,b). These attempts could only model the retina in silicon, however, and did not provide a realistic implementation for practical use. In response, the neuromorphic community focused on the operating principle of the neurobiological architecture rather than modeling the overall sensory system. Specifically, this problem could be solved by realizing the difference between temporal and spatial contrasts. With the developments in AER, pixels could operate individually as processing units and report any deviations in temporal contrast. The spiking output is similar to the action potentials generated by ganglion cells and consequently most retinomorphic sensors now use AER communication (Posch et al., 2014).

Tobi Delbruck built on the idea of adaptive photoreceptor circuits developed in Delbruck and Mead (1994) and introduced strategies for enhancing retinomorphic sensors. The 128 × 128 pixel Dynamic Vision Sensor (DVS) can be considered as the product of several improvements through (Lichtsteiner et al., 2004, 2006; Lichtsteiner and Delbruck, 2005) where the concepts of differentiating ON/OFF events with respect to lumosity change and relative lumosity change were implemented. DVS established a benchmark in neuromorphic vision sensing with its AER- based approach in which each individual pixel processed the normalized time derivative of the sensed light and provided an output in the form of spikes of the pixel addresses that detect lumosity change. As an alternative approach, frame-based temporal detection imagers (Gottardi et al., 2009; Cottini et al., 2013) were developed. The operating principle of these imagers was based on integration of the photocurrent between successive frames and computing of the difference between them. However these implementations have a limited speed response and a low-dynamic range, 100 dB for Gottardi et al. (2009) and 52 dB for Cottini et al. (2013). With features such as sub-millisecond precision, dynamic range >120 dB, and low power consumption of 23 mW, DVS was a path-breaking discovery and was used in various robotic and real-time systems (Rüedi et al., 2003; Drazen et al., 2011; Brandli et al., 2013).

With DVS, a benchmark was established for essential characteristics that a neuromorphic vision sensor should possess and gave a clear direction for further research in vision sensing. Developing on the basic idea of DVS, (Serrano-Gotarredona and Linares-Barranco, 2013) enhanced the capabilities of DVS by improving the contrast sensitivity by one order of magnitude (down to 1.5%) and reducing power consumption to 4 mW and fixed pattern noise to 0.9% and thus the overall pixel size of the sensor down to 30 × 31 μm^2^ per pixel. The QVGA (304 × 240 pixel) ATIS (Asynchronous Time-based Image Sensor) by Posch et al. (2011), implemented PWM (Pulse Width Modulation) based intensity readout that improved the dynamic range (143–125 dB) at the cost of increased pixel area of 30 × 30 μm^2^. The inclusion of DVS pixel and PWM intensity readout triples the sensor output data. Brandli et al. (2014) proposed a hybrid approach between frame-based and frame-free vision sensing. The 240 × 180 pixel DAVIS (Dynamic and Active-pixel Vision Sensor) arose in part from contributions by Berner et al. (2013), in which the concept of integrating a synchronous active-pixel sensor with asynchronous DVS pixels was implemented.

The successful implementation of DAVIS set another benchmark that will inspire future work toward neuromorphic vision sensors that provides spatial details of static scenes while also responding to dynamic temporal changes with minimum latency. This will also drive efforts to improve the fill factor and dynamic range.

## Neuromorphic auditory sensors

The conventional method of sensing auditory signals is sampling the data continuously for auditory input at an application specific Nyquist frequency. This data undergoes Analog-to-Digital Conversion (ADC) and further digital processing to generate auditory frames. There is a significant power cost for high resolution ADC and digital processing of auditory frames. Although, this sampling rate can be altered dynamically to reduce power consumption, there is a risk of losing critical information due to low sampling rate. For applications such as auditory scene analysis, it is necessary that such sensors use less power and generate sparse data (Liu et al., 2014).

Lyon and Mead, proposed an auditory sensor in Lyon and Mead (1988) that models the human cochlea using aVLSI. This work addressed key concepts like automatic gain control, the use of cascaded second-order resonant lowpass filters and the necessary quality factors for auditory applications such as delay, high-gain pseudoresonance, and sharp roll-off. Although, these researchers implemented the bio-physics of both outer and inner hair cells of the basilar membrane, this analog cochlea model did not incorporate any biasing circuits for process, voltage, and temperature variations. However, it was further improved by addressing issues such as device mismatch, stability, and dynamic range (Watts et al., 1992). By introducing “overlapping cochlear cascades” (Sarpeshkar et al., 1998), established a novel approach to the design of an aVLSI cochlea with dynamic range of 61 dB that consumes 0.5 mW. These early works underpinned further studies in silicon cochlea design.

Crucially, the efforts in silicon cochlea research led to the development of an auditory processor for cochlear implants that operated on minimal power. Sarpeshkar and his colleagues extending their work in Wang et al. (1997) and Baker et al. (2003), and taking inspiration from other contributions (Baker and Sarpeshkar, 2003; Salthouse and Sarpeshkar, 2003), developed an ultra-low power auditory processor for a bionic ear. This processor can theoretically operate on a 100 mAh rechargeable battery for several years and features automatic gain control and microphone pre-amplifier audio front end, so that the processor converts the input signal to the desired dynamic range. The digital output of the processor ensures its independence from voltage and temperature variations. It operates over 16 channels that are comprised of independently programmable bandpass filters. Sarpeshkar et al. (2005) claimed that such processors can be applied in systems needing low-powered portable speech recognition front ends.

Along with cochlear implants, auditory scene analysis is a crucial application for the silicon cochlea. The implementation of AER to communicate output spikes stimulated further research on developing a spatial auditory sensor. Building on the silicon cochlea described by Van Schaik et al. (1996) and a neuromorphic front end MEMS (Micro-Electro-Mechanical Systems) microphone explained in Van Schaik and Shamma (2004), Chan et al. (2007) developed AER EAR, a matched pair of silicon cochlea with an AER interface. This auditory sensor models the basilar membrane bio-physics by cascading low-pass filters to provide output over 32 channels. The simplified inner hair cell circuit and spiking neurons ensured sparse asynchronous output; the design was tested for localization applications by computing the interaural time delay between the matched pair of silicon cochleas (Yu et al., 2009).

Further improvements including microphone pre-amplifiers and per-channel capability led to the development of AEREAR2 (Liu et al., 2014), a 64 channel binaural audition sensor that set a benchmark in neuromorphic audition. By integrating local Digital-to-Analog Converters (DAC) that enable the quality factors of individual channels to be adjusted, this sensor overcomes most of the drawbacks of AEREAR. With improved dynamic range, binaural structure, integrated microphone preamplifiers, and biasing circuits for stability against voltage and temperature variance, this sensor provides precise timing of spikes over a USB interface. This approach was used in complex applications like speaker identification (Li et al., 2012). A thorough comparison between conventional cross-correlation approaches and spike-based sound localization algorithms shows that event-driven methods are about 40 times less computationally demanding (Liu et al., 2014). Even more precise and efficient neuromorphic auditory systems will be developed by applying interesting approaches such as spike based audio front ends described in Koickal et al. (2011).

## Neuromorphic olfactory sensors

The development of artificial olfaction devices started with Moncrieff (1961), who applied mechanical concepts to measuring and determining odors. Since the development of the electronic nose (Persaud and Dodd, 1982), emphasis has been placed on developing olfactory sensors that are portable and precise. Conventional olfactory sensors are large with restricted portability and also impede reliability as the chemical constituents in a target gas vary rapidly. These factors along with high manufacturing costs have largely restricted the use of such sensors to laboratory experiments and industries (Chiu and Tang, 2013).

The electronic nose has benefited from CMOS and MEMS technologies, advanced pattern matching methods and new sensing materials (Gardner et al., 2010). Biologically based olfaction systems have inspired a general structure for electronic nose systems that are composed of a sensor array, signal conditioning circuitry, and a pattern recognition unit (Raman et al., 2008). By applying neuromorphic concepts, improvements were made to this structure by integrating all these units on a single chip and implementing neural networks for pattern recognition. Thomas Koickal and colleagues made a notable contribution to developing an adaptive neuromorphic olfaction chip. The CB polymer sensor array used in that system was fabricated using the AMS 0.6 μm CMOS process (Koickal et al., 2007). A novel design was implemented to cancel the baseline sensor variations due to sensor poisoning and variation in operating current specifications across different sensors. The neuromorphic implementation simplified the odor detection especially in the presence of background odor signals. This design proved to be a technological benchmark to stimulate further study in neuromorphic olfaction by introducing features like on-chip Spike Timing Dependent Plasticity (STDP) learning, reduced power consumption, and temporal spiking signals output. Covington et al. (2007) developed a biomimetic mucosa that can generate spatio-temporal output but improvements were needed for reduced response times and odor delivery channel size in this design.

Researchers have focused on implementing the critical olfaction characteristics rather than emulating the entire biological olfactory pathway. A 4 × 4 tin oxide gas sensor array was designed (Ng et al., 2011) such that each row forms a group of sensors showing similar drift behavior. It is possible to detect a wide range of chemical gases by assigning the same catalyst to each group of sensors. The firing delay in the spiking output from these sensors generates a unique sequence of drift-insensitive spikes (Ng et al., 2009). This output represents a signature for a specific gas which is determined by matching it in a library of 2-D spatio-temporal spike signatures. This approach reduces the computation challenges involved in pattern matching (Ng et al., 2010). The entire gas recognition circuit is fabricated and implemented on a single CMOS chip and the power consumption is as low as 6.6 mW with 94.9% identification accuracy.

E-Nose described in Tang et al. (2011), consists of a conducting polymer sensor chip, interface circuitry, ADC, and a microprocessor with a pattern recognition algorithm and an associated memory module. The output of this chip is a unique signature of the target gas, but the inclusion of a pattern matching algorithm instead of a neural network makes this approach computationally expensive. This approach was also used by Tang et al. (2010) to identify and classify fruity odors and led to the development of a spiking neural network chip that implements the Spike Timing Dependent Plasticity (STDP) learning rule (Hsieh and Tang, 2012). The sensor array used for sampling odor data is a commercial electronic nose (Cyranose 320). This work focused on the backend computation to identify odor and the developed chip can identify three different odors concurrently. The average power consumption is as low as 3.6 μW and mean testing accuracy is 87.59%. NEUROCHEM is an important project lead by European universities that is focused on developing a large sensor array for neuromorphic olfactory systems (Bernabei et al., 2012). This conductive polymer sensor array mimics the essential characteristics of biological Odor Receptor Neurons (ORNs) including redundancy and sensitivity to a wide range of volatile compounds.

The neuromorphic olfactory sensor literature indicates that there is considerable scope for improving these sensors. The CMOS chip by Thomas Koickal is a notable contribution in neuromorphic olfaction and can be considered as a highly cited research contribution in neuromorphic olfaction. Although, there are several shortcomings in this implementation, it proposes a novel architecture for olfactory sensors. Improvements proposed in Ng et al. (2011) are promising if the response time can be improved further. Chiu and Tang (2013) also exposes gaps in interfacing, signal conditioning, and pattern matching computations in neuromorphic olfaction.

## Trends in sensor fusion application

While neuromorphic sensors offer benefits of low-power consumption and sparse output data generation, the means to process the spike-based data format is still limited. Decades of research in digital image processing and digital signal processing, has led to the development of advanced algorithms and hardware architectures that allowed efficient processing of conventional outputs (e.g., frame-based and audio samples). As techniques for high-level processing of event-based data are still under development, the large-scale application of neuromorphic sensors depends on the introduction of these techniques. However, advanced research in neuromorphic sensors has increased the application scope of these sensors in intelligent embedded systems. Prototypes of neuromorphic vision and auditory sensors evolved into commercial products such as the DVS128 PAER and DAS1. However, most of the systems that make use of neuromorphic sensing implement only a single type of sensor such as vision or auditory. Rajapakse and Acharya (1991) and Bečanović et al. (2005) were among the first who targeted the development of platforms for interfacing multiple neuromorphic sensors. Chan et al. (2012) implemented sensor fusion of Audio-Video (AV) neuromorphic sensors and presented an advanced version of the Koala robot that was first developed by Bečanović et al. (2005) for object tracking. Development of neuromorphic processing boards under large scale projects such as CAVIAR, BrainScaleS and SpiNNaker, promotes the idea of sensor fusion, and data correlation. By utilizing concepts like the spiking Deep Belief Network (DBN; O'Connor et al., 2013), the idea of multi-sensor neuromorphic systems can be brought to fruition. Such systems can have numerous applications in fields such as robotics, biosecurity and environmental monitoring to name a few.

## Conclusion

In this paper, we have reviewed some of the most significant research contributions toward the improvement of neuromorphic vision, auditory, and olfactory sensors. The distinctive properties of neuromorphic sensors, such as sparse data output and low power consumption have led to extensive research and commercialization. The concept of developing neuromorphic sensors by emulating neuro-biological sensing in silicon has been progressing for many years. More recently, path-breaking research in biological sensing has provided an impetus to developments in neuromorphic sensing, especially in vision and auditory sensors. Pioneering contributions such as DVS and DAVIS, and AEREAR2 have provided considerable progress toward a sensor design that simulates neuro-biological vision and auditory sensing. Accordingly, these have led to the development of several applications for these sensors aiming at replacing conventional sensors in vision and audition. What is lacking is research that provides benchmarks for olfactory sensor implementation and its performance evaluation. Subsequently, future development in neuromorphic sensing should focus on the correlation of inputs from different sensors and efficient pre-processing. With this review we have identified challenges for future research on neuromorphic olfaction, building on the advancements made in vision and audition. In addition to neuromorphic olfaction, future research directions should target neuromorphic sensing of parameters such as pressure, vibration, thermal, and magnetic field as well as their intercorrelated sensor fusion functions which would be ideal for applications such as the Internet of Things (IoT).

## Author contributions

AV has contributed more than 75% to this article and is the first author. AO is the principal supervisor of AV and has contributed to this article as a second author. AR is the co-supervisor of AV and has contributed toward content editing and refining. AR is the third author of this article.

### Conflict of interest statement

The authors declare that the research was conducted in the absence of any commercial or financial relationships that could be construed as a potential conflict of interest.

## References

[B1] Baker M. W. Lu T. K. Salthouse C. D. Sit J.-J. Zhak S. Sarpeshkar R. (2003). A 16-channel analog VLSI processor for bionic ears and speech-recognition front ends, in Custom Integrated Circuits Conference, 2003. Proceedings of the IEEE 2003 (San Jose, CA: IEEE).

[B2] Baker M. W. Sarpeshkar R. (2003). A low-power high-PSRR current-mode microphone preamplifier. IEEE J. Solid State Circuits 38, 1671–1678. 10.1109/JSSC.2003.817255

[B3] Bečanović V. Hosseiny R. Indiveri G. (2005). Object tracking using multiple neuromorphic vision sensors, in RoboCup 2004: Robot Soccer World Cup VIII, eds Nardi D. Riedmiller M. Sammut C. Santos-Victor J. (Berlin: Springer), 426–433.

[B4] Bernabei M. Persaud K. C. Pantalei S. Zampetti E. Beccherelli R. (2012). Large-scale chemical sensor array testing biological olfaction concepts. Sens. J. IEEE 12, 3174–3183. 10.1109/JSEN.2012.2207887

[B5] Berner R. Brandli C. Yang M. Liu S.-C. Delbruck T. (2013). A 240 × 180 10mW 12us latency sparse-output vision sensor for mobile applications, in IEEE Symposium on VLSI Circuits (VLSIC), 2013 (Kyoto).

[B6] Boahen K. (2000). Point-to-point connectivity between neuromorphic chips using address events. IEEE Trans. Circuits Syst. II Anal. Digital Signal Process. 47, 416–434. 10.1109/82.842110

[B7] Brandli C. Berner R. Yang M. Liu S.-C. Delbruck T. (2014). A 240 × 180 130 dB 3 μs latency global shutter spatiotemporal vision sensor. IEEE J. Solid State Circuits 49, 2333–2341. 10.1109/JSSC.2014.2342715

[B8] Brandli C. Mantel T. A. Hutter M. Höpflinger M. A. Berner R. Siegwart R.. (2013). Adaptive pulsed laser line extraction for terrain reconstruction using a dynamic vision sensor. Front. Neurosci. 7:275. 10.3389/fnins.2013.00275 24478619 PMC3894568

[B9] Chan V. Liu S.-C. Van Schaik A. (2007). AER EAR: a matched silicon cochlea pair with address event representation interface. IEEE Trans. Circuits Syst. I Regul. Pap. 54, 48–59. 10.1109/TCSI.2006.887979

[B10] Chan V. Y.-S. Jin C. T. van Schaik A. (2012). Neuromorphic audio–visual sensor fusion on a sound-localizing robot. Front. Neurosci. 6:21. 10.3389/fnins.2012.00021 22347165 PMC3274764

[B11] Chicca E. Stefanini F. Bartolozzi C. Indiveri G. (2014). Neuromorphic electronic circuits for building autonomous cognitive systems. Proc. IEEE 102, 1367–1388. 10.1109/JPROC.2014.2313954

[B12] Chiu S.-W. Tang K.-T. (2013). Towards a chemiresistive sensor-integrated electronic nose: a review. Sensors 13, 14214–14247. 10.3390/s131014214 24152879 PMC3859118

[B13] Cottini N. Gottardi M. Massari N. Passerone R. Smilansky Z. (2013). A 33 μW 64 × 64 pixel vision sensor embedding robust dynamic background subtraction for EVENT detection and scene interpretation. IEEE J.Solid State Circuits 48, 850–863. 10.1109/JSSC.2012.2235031

[B14] Covington J. A. Gardner J. W. Hamilton A. Pearce T. Tan S.-L. (2007). Towards a truly biomimetic olfactory microsystem: an artificial olfactory mucosa. IET Nanobiotechnol. 1, 15–21. 10.1049/iet-nbt:20060015 17428120

[B15] Delbruck T. Mead C. (1994). Adaptive photoreceptor with wide dynamic range. Circuits and Systems, 1994, in IEEE International Symposium on ISCAS'94, 1994 (London: IEEE).

[B16] Douglas R. Mahowald M. Mead C. (1995). Neuromorphic analogue VLSI. Annu. Rev. Neurosci. 18, 255–281. 10.1146/annurev.ne.18.030195.001351 7605063

[B17] Drazen D. Lichtsteiner P. Häfliger P. Delbrück T. Jensen A. (2011). Toward real-time particle tracking using an event-based dynamic vision sensor. Exp. Fluids 51, 1465–1469. 10.1007/s00348-011-1207-y

[B18] Gardner J. W. Guha P. K. Udrea F. Covington J. (2010). CMOS interfacing for integrated gas sensors: a review. Sens. J. IEEE 10, 1833–1848. 10.1109/JSEN.2010.2046409

[B19] Gottardi M. Massari N. Jawed S. A. (2009). A 100 μW 128 × 64 pixels contrast-based asynchronous binary vision sensor for sensor networks applications. IEEE J. Solid-State Circuits 44, 1582–1592. 10.1109/JSSC.2009.2017000

[B20] Hasler J. Marr B. (2013). Finding a roadmap to achieve large neuromorphic hardware systems. Front. Neurosci. 7:118. 10.3389/fnins.2013.00118 24058330 PMC3767911

[B21] Hsieh H.-Y. Tang K.-T. (2012). VLSI implementation of a bio-inspired olfactory spiking neural network. IEEE Trans. Neural Netw. Learn. Syst. 23, 1065–1073. 10.1109/TNNLS.2012.2195329 24807133

[B22] Indiveri G. Horiuchi T. K. (2011). Frontiers in neuromorphic engineering. Front. Neurosci. 5:118. 10.3389/fnins.2011.00118 22013408 PMC3189639

[B23] Koickal T. J. Hamilton A. Tan S. L. Covington J. Gardner J. W. Pearce T. C. (2007). Analog VLSI circuit implementation of an adaptive neuromorphic olfaction chip. IEEE Trans. Circ. Syst. I Regul. Pap. 54, 60–73. 10.1109/TCSI.2006.888677

[B24] Koickal T. J. Latif R. Gouveia L. Mastropaolo E. Wang S. Hamilton A. (2011). Design of a spike event coded RGT microphone for neuromorphic auditory systems, in IEEE International Symposium on Circuits and Systems (ISCAS), 2011 (Rio de Janeiro: IEEE).

[B25] Li C.-H. Delbruck T. and Liu, S.-C. (2012). Real-time speaker identification using the AEREAR2 event-based silicon cochlea, in IEEE International Symposium on Circuits and Systems (ISCAS), 2012 (Seoul: IEEE).

[B26] Lichtsteiner P. Delbruck T. (2005). A 64 × 64 AER logarithmic temporal derivative silicon retina. Res. Microelectr. Electr. 2, 202–205. 10.1109/RME.2005.1542972

[B27] Lichtsteiner P. Delbruck T. Kramer J. (2004).Improved ON/OFF temporally differentiating address-event imager, Proceedings of the 11th IEEE International Conference on Electronics, Circuits and Systems, 2004. ICECS 2004 (Tel-Aviv: IEEE).

[B28] Lichtsteiner P. Posch C. Delbruck T. (2006). A 128 × 128 120dB 30mW asynchronous vision sensor that responds to relative intensity change, in IEEE International Conference on Solid State Circuits, 2006 (San Francisco, CA: IEEE).

[B29] Lichtsteiner P. Posch C. Delbruck T. (2008). A 128 × 128 120 dB 15 μs latency asynchronous temporal contrast vision sensor. IEEE J. Solid State Circuits 43, 566–576. 10.1109/JSSC.2007.914337

[B30] Liu S.-C. Delbrück T. Indiveri G. Whatley A. Douglas R. (2015). Event-Based Neuromorphic Systems. Chichester: John Wiley & Sons.

[B31] Liu S.-C. van Schaik A. Minch B. Delbruck T. (2014). Asynchronous binaural spatial audition sensor with 2 64 4 Channel Output. IEEE Trans. Biomed. Circuits Syst. 8, 453–464. 10.1109/TBCAS.2013.2281834 24216772

[B32] Lyon R. F. Mead C. (1988). An analog electronic cochlea. IEEE Trans. Acoust. Speech Signal Process. 36, 1119–1134. 10.1109/29.1639

[B33] Mahowald M. A. Mead C. (1991). The silicon retina. Sci. Am. 264, 76–82. 10.1038/scientificamerican0591-76 2052936

[B34] Mead C. (1990). Neuromorphic electronic systems. Proc. IEEE 78, 1629–1636. 10.1109/5.58356

[B35] Moncrieff R. W. (1961). An instrument for measuring and classifying odors. J. Appl. Physiol. 16, 742–749. 13771984 10.1152/jappl.1961.16.4.742

[B36] Ng K. T. Boussaid F. Bermak A. (2010). A frequency-based signature gas identification circuit for SnO_2_ gas sensors, in Proceedings of IEEE International Symposium on Circuits and Systems (ISCAS), 2010 (Paris: IEEE).

[B37] Ng K. T. Boussaid F. Bermak A. (2011). A CMOS single-chip gas recognition circuit for metal oxide gas sensor arrays. IEEE Trans. Circ. Syst. I Regul. Pap. 58, 1569–1580. 10.1109/TCSI.2011.2143090

[B38] Ng K. T. Guo B. Bermak A. Martinez D. Boussaid F. (2009). Characterization of a logarithmic spike timing encoding scheme for a 4 × 4 tin oxide gas sensor array, in IEEE Sensors, 2009 (Christchurch: IEEE).

[B39] O'Connor P. Neil D. Liu S.-C. Delbruck T. Pfeiffer M. (2013). Real-time classification and sensor fusion with a spiking deep belief network. Front. Neurosci. 7:178. 10.3389/fnins.2013.00178 24115919 PMC3792559

[B40] Persaud K. Dodd G. (1982). Analysis of discrimination mechanisms in the mammalian olfactory system using a model nose. Nature 99, 352–355. 10.1038/299352a0 7110356

[B41] Posch C. Matolin D. Wohlgenannt R. (2011). A QVGA 143 dB dynamic range frame-free PWM image sensor with lossless pixel-level video compression and time-domain CDS. IEEE J. Solid State Circuits 46, 259–275. 10.1109/JSSC.2010.2085952

[B42] Posch C. Serrano-Gotarredona T. Linares-Barranco B. Delbruck T. (2014). Retinomorphic event-based vision sensors: bioinspired cameras with spiking output. Proc. IEEE 102, 1470–1484. 10.1109/JPROC.2014.2346153

[B43] Rajapakse J. Acharya R. (1991). Neuromorphic model for information fusion, in IEEE International Conference on Acoustics, Speech, and Signal Processing, 1991. ICASSP-91. (Toronto).

[B44] Raman B. Hertz J. L. Benkstein K. D. Semancik S. (2008). Bioinspired methodology for artificial olfaction. Anal. Chem. 80, 8364–8371. 10.1021/ac8007048 18855409 PMC2583168

[B45] Rüedi P.-F. Heim P. Kaess F. Grenet E. Heitger F. Burgi P.-Y. (2003). A 128 × 128 pixel 120-dB dynamic-range vision-sensor chip for image contrast and orientation extraction. IEEE J. Solid State Circuits 38, 2325–2333. 10.1109/JSSC.2003.819169

[B46] Salthouse C. D. Sarpeshkar R. (2003). A practical micropower programmable bandpass filter for use in bionic ears. IEEE J. Solid State Circuits 38, 63–70. 10.1109/JSSC.2002.806286

[B47] Sarpeshkar R. (1998). Analog versus digital: extrapolating from electronics to neurobiology. Neural Comput. 10, 1601–1638. 10.1162/089976698300017052 9744889

[B48] Sarpeshkar R. (2006). Brain power-borrowing from biology makes for low power computing [bionic ear]. Spectr. IEEE 43, 24–29. 10.1109/MSPEC.2006.1628504

[B49] Sarpeshkar R. Lyon R. F. Mead C. (1998). A low-power wide-dynamic-range analog VLSI cochlea, in Neuromorphic Systems Engineering (Norwell, MA: Kluwer Academi), 49–103.

[B50] Sarpeshkar R. Salthouse C. Sit J.-J. Baker M. W. Zhak S. M. Lu T. K.-T.. (2005). An ultra-low-power programmable analog bionic ear processor. IEEE Trans. Biomed. Eng. 52, 711–727. 10.1109/TBME.2005.844043 15825873

[B51] Serrano-Gotarredona T. Linares-Barranco B. (2013). A 128 128 1.5% contrast sensitivity 0.9% FPN 3 μs latency 4 mW asynchronous frame-free dynamic vision sensor using transimpedance preamplifiers. IEEE J. Solid-State Circuits 48, 827–838. 10.1109/JSSC.2012.2230553

[B52] Tang K.-T. Chiu S.-W. Chang M.-F. Hsieh C.-C. and Shyu, J.-M. (2011). A low-power electronic nose signal-processing chip for a portable artificial olfaction system. IEEE Trans. Biomed. Circuits Syst. 5, 380–390. 10.1109/TBCAS.2011.2116786 23851952

[B53] Tang K.-T. Chiu S.-W. Pan C.-H. Hsieh H.-Y. Liang Y.-S. and Liu, S.-C. (2010). Development of a portable electronic nose system for the detection and classification of fruity odors. Sensors 10, 9179–9193. 10.3390/s101009179 22163403 PMC3230968

[B54] Tenore F. Etienne-Cummings R. (2011). Neuromorphic Electronic Design, in Biohybrid Systems, ed Jung R. (Weinheim: Wiley-VCH Verlag GmbH & Co. KGaA), 31–51.

[B55] Van Schaik A. Fragnière E. Vittoz E. (1996). Improved silicon cochlea using compatible lateral bipolar transistors. Adv. Neural Inf. Process. Syst. 8, 671–677.

[B56] Van Schaik A. Shamma S. (2004). A neuromorphic sound localizer for a smart MEMS system. Analog Integr. Circuits Signal Process. 39, 267–273. 10.1023/B:ALOG.0000029662.37528.c7

[B57] Wang R. J. W. Sarpeshkar R. Jabri M. Mead C. (1997). A low power analog front-end module for cochlear implants, in Presented at the XVI World Congress on Otorhinolaryngology (Sydney).

[B58] Watts L. Kerns D. Lyon R. F. Mead C. (1992). Improved implementation of the silicon cochlea. IEEE J. Solid State Circuits 27, 692–700. 10.1109/4.133156

[B59] Yu T. Schwartz A. Harris J. Slaney M. Liu S.-C. (2009). Periodicity detection and localization using spike timing from the AER EAR Circuits, R and Systems, 2009, in IEEE International Symposium on ISCAS 2009 (Taipei: IEEE).

[B60] Zaghloul K. A. Boahen K. (2004a). Optic nerve signals in a neuromorphic chip I: outer and inner retina models. IEEE Trans. Biomed. Eng. 51, 657–666. 10.1109/TBME.2003.821039 15072220

[B61] Zaghloul K. A. Boahen K. (2004b). Optic nerve signals in a neuromorphic chip II: testing and results. IEEE Trans.Biomed. Eng. 51, 667–675. 10.1109/TBME.2003.821040 15072221

